# Mp*WIP* regulates air pore complex development in the liverwort *Marchantia polymorpha*

**DOI:** 10.1242/dev.144287

**Published:** 2017-04-15

**Authors:** Victor A. S. Jones, Liam Dolan

**Affiliations:** Department of Plant Sciences, University of Oxford, Oxford OX1 3RB, UK

**Keywords:** *Marchantia polymorpha*, Air pore complex, WIP protein

## Abstract

The colonisation of the land by plants was accompanied by the evolution of complex tissues and multicellular structures comprising different cell types as morphological adaptations to the terrestrial environment. Here, we show that the single WIP protein in the early-diverging land plant *Marchantia polymorpha* L. is required for the development of the multicellular gas exchange structure: the air pore complex. This 16-cell barrel-shaped structure surrounds an opening between epidermal cells that facilitates the exchange of gases between the chamber containing the photosynthetic cells inside the plant and the air outside. Mp*WIP* is expressed in cells of the developing air pore complex and the morphogenesis of the complex is defective in plants with reduced Mp*WIP* function. The role of WIP proteins in the control of different multicellular structures in *M. polymorpha* and the flowering plant *Arabidopsis thaliana* suggests that these proteins controlled the development of multicellular structures in the common ancestor of land plants. We hypothesise that *WIP* genes were subsequently co-opted in the control of morphogenesis of novel multicellular structures that evolved during the diversification of land plants.

## Abstract (precis)

**Highlighted Article:** The Mp*WIP* gene controls the development of the air pore complex – a multicellular structure that increases CO_2_ uptake – in the early-diverging land plant *Marchantia polymorpha*.

## INTRODUCTION

Morphological diversity increased dramatically after plants colonised the land some time before 460 million years ago (Kenrick and Crane, 1997). The evolution of unicellular and multicellular structures with specialised functions in the outermost cell layer – the epidermis – provided plants with the means to increase the surface area over which CO_2_ uptake from the atmosphere occurred, and to extract water and inorganic nutrients from the early soil. Some specialised epidermal structures are present in all extant lineages of land plants. For example, tip-growing rhizoids and root hairs emerge from the epidermis to provide anchorage and to take up water and nutrients from the soil (Jones and Dolan, 2012). The phylogenetic distribution of others is more restricted; stomata, valves in the epidermis consisting of two specialised guard cells that open and close to regulate gas exchange, develop in all land plant lineages except the early diverging Marchantiophyta (liverworts). In one group of liverworts, the Marchantiidae, the evolution of complex tissues has been accompanied by an independent evolution of a multicellular epidermal structure that facilitates gas exchange: the air pore complex (Crandall-Stotler et al., 2009). We report here that the zinc-finger protein MpWIP is necessary for the morphogenesis of the air pore complex in the epidermis of *Marchantia polymorpha*.

## RESULTS AND DISCUSSION

### A gain-of-function mutation in Mp*WIP* causes defective development of the dorsal epidermis

**Fig. 1.**
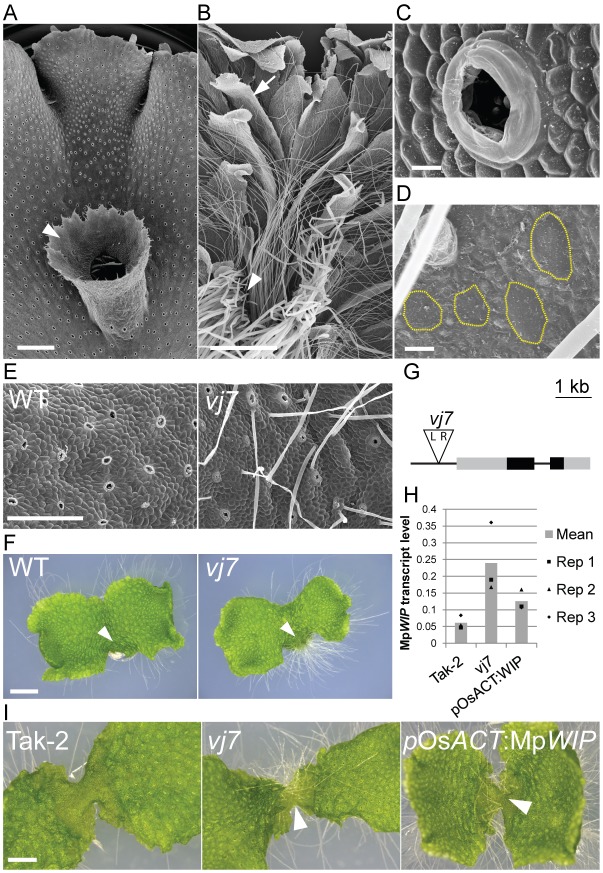
**A gain-of-function mutant of Mp*WIP* develops ectopic rhizoids on the dorsal surface.** (A) Air pores and gemma cups (arrowhead) are produced on the dorsal thallus surface. Scale bar: 1 mm, apex at the top. (B) Scales (arrow) and rhizoids (arrowhead) are produced on the ventral thallus surface. Scale bar: 1 mm, apex at the top. (C) Detail of air pore complex. Scale bar: 20 μm. (D) Detail of ventral rhizoid patch. Cells that will develop into rhizoids (yellow outlines) are separated by non-rhizoid cells. Scale bar: 20 μm. (E) Rhizoids develop on the dorsal surface of older parts of the mature thallus of *vj7* but not wild type at 43 days. Scale bar: 500 µm. (F) Sporelings of *vj7* produce rhizoids on the oldest part of the dorsal thallus surface (arrowhead). This region of wild-type sporelings lacks rhizoids at 28 days. Scale bar: 2 mm. (G) The T-DNA insertion that co-segregates with the mutant phenotype in *vj7* is located 5′ to Mp*WIP*. Boxes represent exons: black, CDS, grey, untranslated regions. (H) Mp*WIP* transcript levels are greater in mutant *vj7* and *_pro_*Os*ACT:*Mp*WIP* than in wild-type Tak-2 in 14-day-old gemmalings. (I) Expression of Mp*WIP* driven by the constitutive promoter *_pro_*Os*ACT* causes the development of ectopic rhizoids (arrowheads), as in mutant *vj7* in 10-day-old gemmalings. Scale bar: 1 mm.

To identify genetic mechanisms controlling the development of specialised morphological structures that operated in the earliest land plants, we screened for mutants with defects in the development of epidermal structures in the liverwort *Marchantia polymorpha*, a member of one of the earliest diverging groups of land plants. Multicellular air pore complexes, gemma cups and gemmae develop on the dorsal epidermis of *M. polymorpha* (Fig. 1A,C), while unicellular rhizoids and multicellular membranous outgrowths (scales) develop on the ventral epidermis (Fig. 1B,D). In a screen of T-DNA insertion mutants (Honkanen et al., 2016), we isolated a mutant, *vj7*, that develops rhizoids from the epidermal cells of the mature dorsal epidermis (3.76 rhizoids/mm^2^, *n*=5); rhizoids do not develop on the dorsal epidermis of the wild type (Fig. 1E,F). We crossed this mutant to the wild-type Tak-1 to determine its inheritance; of 293 F1 plants scored, 131 expressed the mutant phenotype and 162 the wild-type phenotype (segregation ratio 1:1.24, χ_2_ *P*=0.07), demonstrating that the dorsal rhizoid phenotype is controlled by a single Mendelian locus. We located a T-DNA insertion in this line and genotyped 106 of the above F1 offspring for its presence. Seventy-four F1s displayed the mutant phenotype and possessed the insertion, while the remaining 34 were wild type and lacked the insertion, indicating that this insertion is linked to the mutant phenotype. The insertion lies 764 bp upstream of the transcriptional start site of a gene encoding a member of the WIP zinc-finger protein family, MpWIP (GenBank: KX645870) (Figs 1G, S1, S2).

We hypothesised that the T-DNA insertion in *vj7* would impact the transcription of the Mp*WIP* gene 3′ from the T-DNA right border. To quantify the effects of this insertion on Mp*WIP* expression, we measured the steady-state levels of Mp*WIP* transcript in the wild-type and mutant *vj7*. Mp*WIP* transcript levels were almost four times higher in *vj7* than in the wild-type Tak-2 (Fig. 1H), consistent with the hypothesis that *vj7* is a gain-of-function Mp*wip* mutant. To independently verify that Mp*WIP* gain of function induces the development of rhizoids on the dorsal surface of *M. polymorpha*, we expressed Mp*WIP* under the control of the constitutively active Os*ACTIN* promoter (*_pro_*Os*ACT:*Mp*WIP*) (Breuninger et al., 2016), and isolated a line in which the level of Mp*WIP* transcript is twice that seen in the wild type (Fig. 1H). Plants of this line developed ectopic rhizoids on the dorsal surface, as observed in *vj7* but not the wild-type Tak-2 (Fig. 1I). This is consistent with the hypothesis that a gain of Mp*WIP* function causes the development of ectopic rhizoids in mutant *vj7*. We conclude that *vj7* is a gain-of-function mutant of Mp*WIP* and designated it Mp*wip-1^GOF^*.

### The Mp*WIP* promoter is active in developing air pores

**Fig. 2.**
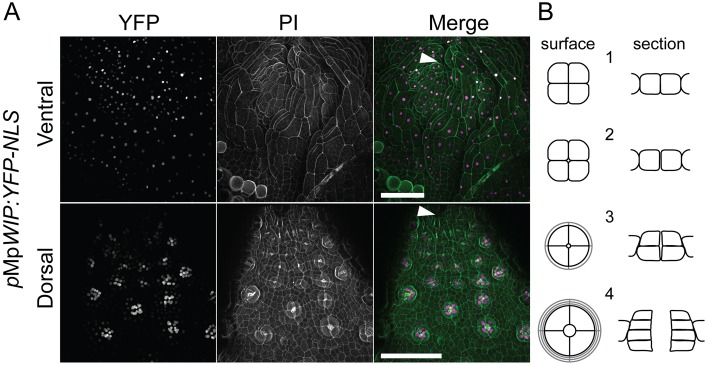
**The Mp*WIP* promoter is active in the ventral apical region and in developing air pores.** (A) Apical region of the ventral and dorsal surface of the thallus of a *_pro_*Mp*WIP:3xYFP-NLS* 9-day-old gemmaling. Scale bars: 100 µm; arrowheads indicate the apex. (B) Schematic of the stages of air pore development. A schizogenous opening develops at the point where four epidermal cells meet (stages 1 and 2). Periclinal divisions then give rise to a stack of rings, each consisting of four cells (stages 3 and 4). Surface view (left) and cross-section (right). Based on data from Apostolakos and Galatis (1985a).

To investigate where the Mp*WIP* promoter is active in the wild type, we expressed *3xYFP-NLS* under the control of a 4.7 kb fragment of genomic DNA upstream of the coding DNA sequence (CDS) of Mp*WIP* (*_pro_*Mp*WIP*:*YFP-NLS*). In plants transformed with *_pro_*Mp*WIP*:*YFP-NLS*, fluorescent protein was detected in cells in the apical region of both the ventral and dorsal sides of the thallus (Fig. 2A). The activity of the promoter in the ventral apical region, where rhizoids initiate, is consistent with a possible role for Mp*WIP* in promoting rhizoid development. On the dorsal side of the thallus, the Mp*WIP* promoter was most active in cells of developing air pore complexes (Fig. 2A), with lower activity in the surrounding epidermal cells. Air pores initiate as schizogenous openings that form in the epidermis at points where four cells meet (Apostolakos and Galatis, 1985a). The four cells surrounding each opening divide periclinally and differentiate to form the multiple tiers of the barrel-shaped air pore (Fig. 2B) (Apostolakos and Galatis, 1985a). Air chambers form below the air pores and consist of schizogenous intercellular cavities in which filaments of photosynthetic cells develop (Apostolakos and Galatis, 1985b; Ishizaki et al., 2013; Mirbel, 1835). Low levels of *_pro_*Mp*WIP* activity were detected in all cells near the apex before air pore differentiation is visible, and this activity increased in the dividing cells of the developing air pore complex. The strong promoter activity in cells of the air pore complexes compared with surrounding cells is first apparent at the four-cell stage, when the cells surrounding the schizogenous opening first enlarge relative to the surrounding epidermal cells (Fig. 2A,B). Strong expression continues during the periclinal divisions that generate the tiered 16-cell air pore complex (Fig. 2A,B). The activity of the Mp*WIP* promoter during the formation of air pore complexes suggested that MpWIP could be involved in their development.

### MpWIP is required for air pore development

**Fig. 3.**
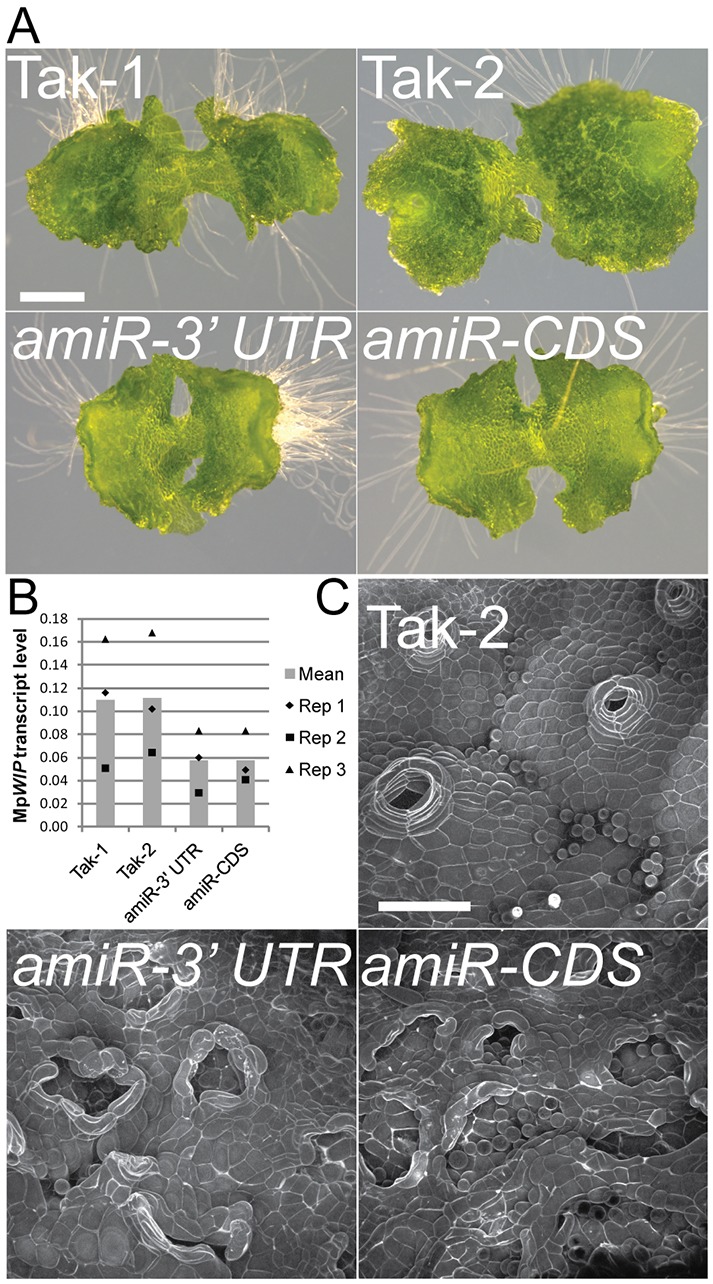
**Reduced Mp*WIP* expression causes defects in air pore development.** (A) The dark-green air chambers seen in the wild type (Tak-1, Tak-2) do not develop in plants transformed with *_pro_*Os*ACT:amiR-*Mp*WIP-3′ UTR*^Mp*miR160*^ or *_pro_*Os*ACT:amiR-*Mp*WIP-CDS*^Mp*miR160*^. Images taken in 10-day-old gemmalings. Scale bar: 1 mm. (B) Mp*WIP* transcript levels are reduced in lines transformed with *_pro_*Os*ACT:amiR-*Mp*WIP-3′ UTR*^Mp*miR160*^ or *_pro_*Os*ACT:amiR-*Mp*WIP-CDS*^Mp*miR160*^. Values are from 10-day-old gemmalings. (C) Plants with reduced Mp*WIP* transcript levels develop air pores with defective morphology, lacking the regular 16-cell air pore complex structure that develops in the wild type (Tak-2). CSLM: images are gemmalings PI stained at 9 days. Scale bar: 100 μm.

To determine whether MpWIP is required for rhizoid or air pore complex development, we generated plants with decreased Mp*WIP* function. We expressed two different artificial microRNAs based on Mp*miR160* (Flores-Sandoval et al., 2016) that target either the 3′ UTR (*amiR-*Mp*WIP-3′ UTR*^Mp*miR160*^) or CDS (*amiR-*Mp*WIP-CDS*^Mp*miR160*^) of Mp*WIP* under the control of *_pro_*Os*ACT*. Steady-state levels of Mp*WIP* transcript are reduced to approximately half wild-type levels in plants transformed with *_pro_*Os*ACT:amiR-*Mp*WIP-3′ UTR*^Mp*miR160*^ or *_pro_*Os*ACT:amiR-*Mp*WIP-CDS*^Mp*miR160*^ (Fig. 3A,B). The formation of the air chambers is delayed or abolished in the Mp*WIP* knockdown lines, and consequently the reticulated pattern of dark-green air chambers characteristic of the wild type is absent (Fig. 3A); this is in contrast to the Mp*wip-1_GOF_* mutant, in which the density of air pore production is similar to the wild type (Fig. S3). Furthermore, the regular 16-cell structure of the wild-type air pore complex does not develop (Fig. 3C). Air pore development begins with the formation of schizogenous openings at the point where four cells meet, exactly as it does in the wild type (Fig. S4A). However, the periclinal divisions that form the tiers of the air pore complex in wild type mostly fail to occur in the knockdown lines. Instead, cells divide anticlinally, forming a single tier of more than four cells surrounding the pore (Fig. S4B). This indicates that reducing the level of Mp*WIP* transcript disrupts air pore morphogenesis after the four-cell stage, consistent with a role for Mp*WIP* in air pore complex and air chamber development suggested by the activity of *_pro_*Mp*WIP* during air pore development (Fig. 2A). We were unable to quantify rhizoid density, but rhizoid development was indistinguishable from the wild type. Together, these data indicate that Mp*WIP* activity is required for the differentiation of air pore complexes, but do not provide evidence that it is necessary for rhizoid development.

### MpWIP may act as a transcriptional repressor

At least one WIP protein, AtNO TRANSMITTING TRACT (AtNTT), binds DNA (Marsch-Martínez et al., 2014). To determine whether MpWIP promotes rhizoid identity and air pore complex development through transcriptional activation or repression, we expressed chimeric dominant repressor and activator versions of MpWIP separately in transgenic plants. To generate the dominant repressor, we fused an SRDX repressive domain (Hiratsu et al., 2003) to the C-terminus of MpWIP; to make the dominant activator, we fused a VP16 activator domain to the C-terminus (Liu and Stewart, 2016; Sadowski et al., 1988; Wilde et al., 1994). Each of these fusion proteins was expressed using the constitutive CaMV 35S promoter (*_pro_35S:*Mp*WIP-SRDX* and *_pro_35S:*Mp*WIP-VP16*). If MpWIP promotes rhizoid and air pore differentiation via transcriptional repression, we predicted that: (1) supernumerary rhizoids would develop on plants that express Mp*WIP-SRDX*, as observed in plants overexpressing Mp*WIP* function (Fig. 1E,F,H,I); and (2) plants expressing Mp*WIP-VP16* would develop a defective air pore phenotype similar to that caused by a loss of Mp*WIP* function in *_pro_*Os*ACT:amiR-*Mp*WIP-3′ UTR*^Mp*miR160*^ and *_pro_*Os*ACT:amiR-*Mp*WIP-CDS*^Mp*miR160*^ lines (Fig. 3A-C).

**Fig. 4.**
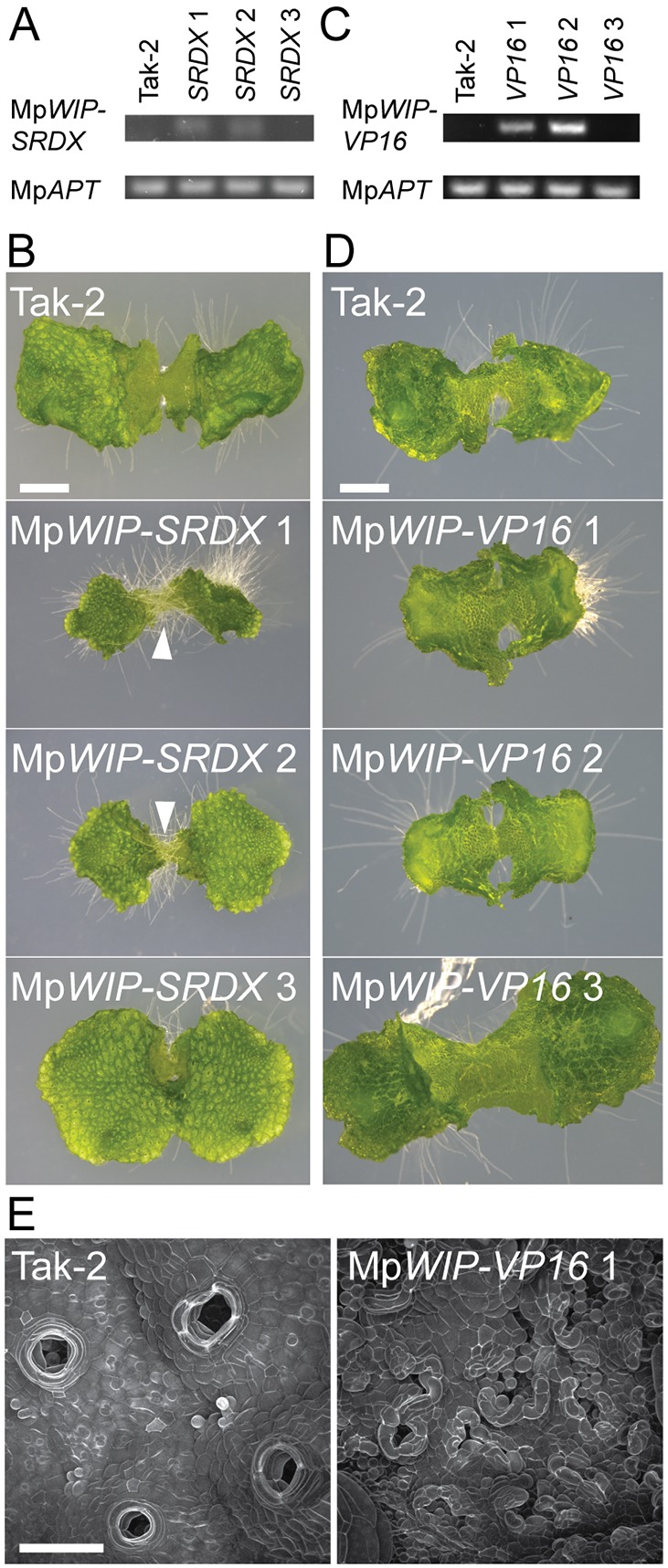
**Expression of the dominant repressor Mp*WIP*-*SRDX* or the dominant activator Mp*WIP-VP16* causes the development of ectopic rhizoids or defective air pores, respectively.** (A) Mp*WIP:SRDX* transcript is detected in lines *_pro_35S:*Mp*WIP:SRDX* 1 and 2 but not in *_pro_35S:*Mp*WIP-SRDX* 3 or Tak-2. Samples are from 12-day-old gemmalings. (B) The lines that express Mp*WIP-SRDX* (*_pro_35S:*Mp*WIP-SRDX* 1 and 2) develop ectopic rhizoids on the dorsal surface (arrowheads). Images are of 12-day-old gemmalings. Scale bar: 2 mm. (C) Mp*WIP-VP16* transcript is detected in lines *_pro_35S:*Mp*WIP-VP16* 1 and 2 but not *_pro_35S:*Mp*WIP-VP16* 3 or Tak-2. Samples are from 10-day-old gemmalings. (D) Air chamber development is defective in lines that express Mp*WIP-VP16* (*_pro_35S:*Mp*WIP-VP16* 1 and 2). Images are of 10-day-old gemmalings. Scale bar: 1 mm. (E) Air pore complex morphology is aberrant in lines that express Mp*WIP-VP16*. CSLM: images are gemmalings PI stained at 10 days. Scale bar: 100 μm.

Plants transformed with *_pro_35S:*Mp*WIP:SRDX* that expressed the transgene (Fig. 4A) developed a dense growth of ectopic rhizoids on the dorsal surface of the thallus, while air pore development was similar to wild type (Fig. 4C). This is similar to the phenotype of the Mp*wip^GOF^* mutant and *_pro_OsACT:*Mp*WIP* line (Fig. 1E,F,I). The expression of a repressive form of Mp*WIP* therefore results in the development of plants that are morphologically similar to plants that overexpress native Mp*WIP*, consistent with the hypothesis that MpWIP is a transcriptional repressor. Plants that express the Mp*WIP-VP16* transgene (Fig. 4C) developed phenotypic defects comparable with those in lines with reduced Mp*WIP* function, where air chamber (Figs 4D and 3A) and air pore complex development are defective (Figs 4E and 3C). This suggests that expression of a form of MpWIP that promotes transcriptional activation has developmental effects similar to a loss of Mp*WIP* function. Therefore, the phenotypes of both Mp*WIP:SRDX* and Mp*WIP:VP16* lines are consistent with the hypothesis that MpWIP promotes the morphogenesis of air pore complexes through transcriptional repression.

We conclude that Mp*WIP* is necessary for the morphogenesis of the multicellular air pore complex in the dorsal epidermis of *M. polymorpha*; air pore morphology is defective in plants with reduced WIP protein activity. *WIP* genes are also required for the development of various multicellular structures in the angiosperm *A. thaliana.* For example, AtNTT is a WIP protein required for the development of the replum, a structure that facilitates dehiscence and seed dispersal from *A. thaliana* fruits (Marsch-Martínez et al., 2014) – cell number is reduced in the repla of At*ntt* mutant fruits compared with wild type. Roots do not form in At*ntt* At*wip4* At*wip5* triple mutants, demonstrating a requirement for these three related WIP proteins in the development of the distal stem cells of the root during embryogenesis (Crawford et al., 2015). Incomplete veins form in At*defectively organised tributaries5* (At*dot5*) mutants, indicating the requirement of the WIP protein AtDOT5 in leaf vein development (Petricka et al., 2008). The demonstration that WIP proteins control the development of different multicellular structures in both early-diverging land plants and angiosperms (the latest-derived land plants) leads us to propose that WIP proteins control the development of multicellular structures in the common ancestor of *M. polymorpha* and *A. thaliana*, a close relative of the earliest land plants. We hypothesise that the subsequent duplication of *WIP* genes and neofunctionalisation of WIP proteins promoted the development of novel multicellular structures that evolved as the morphologies of land plants diversified.

## MATERIALS AND METHODS

### Plasmid construction

The generation of vectors for the constitutive expression of MpWIP, fusion proteins and artificial microRNAs, and of the MpWIP promoter reporter construct, is described in the supplementary Materials and Methods. See Table S1 for oligonucleotide sequences.

### Phylogenetic analysis

Mp*WIP* was aligned with WIP proteins from other land plants and the most similar non-WIP proteins from *M. polymorpha* and *A. thaliana*. This alignment was manually trimmed and used to infer a maximum-likelihood phylogeny. For further details, see the supplementary Materials and Methods.

### Plant material and growth

Tak-1 male and Tak-2 female wild-type accessions (Ishizaki et al., 2008) were used in this study. Mutant *vj7* was isolated in a mutant screen of spores from a cross between Tak-1 and Tak-2 transformed with the T-DNA vector pCambia1300 (Honkanen et al., 2016). Plants were grown as previously described (Honkanen et al., 2016).

### Microscopy

Images were obtained using a Leica M165FC stereomicroscope, Leica M series Plan APO 1.0× objective and Leica DFC310 FX camera. For confocal scanning laser microscopy (CSLM), plants were stained with 15 µM propidium iodide for 15 min, then submerged in water. Images were acquired with a Leica SP5 confocal microscope using a Leica HCX APO 40×/0.80 W U-V-I dipping lens with sequential scans. YFP fluorescence was detected using excitation at 514 nm with an argon laser and emission was measured between 524 and 568 nm using an Acousto-Optic Tunable Filter. PI was excited at 543 nm using a helium-neon laser and emission measured between 568 and 659 nm. Images were processed using FIJI to create brightest-point 3d projections (Schindelin et al., 2012).

For scanning electron microscopy, samples were fixed in dry methanol, critical point dried using a Tousimis Autosamdri-815, mounted on aluminium stubs and coated with a gold/palladium mixture using a Quorum Technologies SC7640 sputter coater. Samples were imaged immediately with a JEOL JSM-5510 SEM.

### Molecular analysis of mutant *vj7* and gene expression analysis

Genomic sequences flanking T-DNA insertions were isolated by TAIL-PCR as previously described (Proust et al., 2016). Genes near the site of the insertion linked to the mutant phenotype in line *vj7* were identified using the blastn algorithm, with 5 kb of genomic sequence 3′ and 5′ to the insertion site as the template, to query an *M. polymorpha* transcriptome (Honkanen et al., 2016). RNA extraction, cDNA synthesis and quantitative PCRs (qPCRs) were carried out as previously described (Breuninger et al., 2016). Mp*ACT* and Mp*APT* were used as reference genes (Saint-Marcoux et al., 2015).
